# Experimental and Numerical Studies of the Temperature Field in a Dielectrophoretic Cell Separation Device Subject to Joule Heating

**DOI:** 10.3390/s24217098

**Published:** 2024-11-04

**Authors:** Yoshinori Seki, Shigeru Tada

**Affiliations:** Department of Applied Physics, National Defense Academy, Hashirimizu 1-10-20, Yokosuka City 239-0802, Kanagawa, Japan; ed22004@nda.ac.jp

**Keywords:** dielectrophoresis, microfluidics, Joule heat, numerical simulation, micro-LIF method

## Abstract

Technologies for rapid and high-throughput separation of rare cells from large populations of other types of cells have recently attracted much attention in the field of bioengineering. Among the various cell separation technologies proposed in the past, dielectrophoresis has shown particular promise because of its preciseness of manipulation and noninvasiveness to cells. However, one drawback of dielectrophoresis devices is that their application of high voltage generates Joule heat that exposes the cells within the device to high temperatures. To further explore this problem, this study investigated the temperature field in a previously developed cell separation device in detail. The temperature rise at the bottom of the microfluidic channel in the device was measured using a micro-LIF method. Moreover, the thermofluidic behavior of the cell separation device was numerically investigated by adopting a heat generation model that takes the electric-field-dependent heat generation term into account in the energy equation. Under the operating conditions of the previously developed cell separation device, the experimentally obtained temperature rise in the device was approximately 20 °C, and the numerical simulation results generally agreed well. Next, parametric calculations were performed with changes in the flow rate of the cell sample solution and the solution conductivity, and a temperature increase of more than 40 °C was predicted. The results demonstrated that an increase in temperature within the cell separation device may have a significant impact on the physiological functions of the cells, depending on the operating conditions of the device.

## 1. Introduction

Recently, dielectrophoresis (DEP) has been attracting attention as a technique for separating rare cells from a large number of cells [1,2,3]. DEP is a phenomenon in which dielectric microparticles placed in a nonuniform electric field move under the force along the gradient of the electric field [4], and this principle can be used to manipulate cells noninvasively and with high precision. The application of the DEP principle utilizing an A.C. electric field makes it possible to selectively manipulate biological microparticles, such as cells, with high precision according to their inherent electrical properties. This capability contributes to the development of critical fundamental technologies in life sciences, such as cell separation, concentration, purification, and manipulation technologies. Therefore, there is a strong demand for the development of rapid and high-precision cell separation technologies that leverage the advantages of DEP [5,6,7].

Among the many forms of DEP-based microdevices, electrode-based dielectrophoresis (eDEP) devices are distinguished by the large electric field gradient generated within the microdevice by microelectrodes [8,9,10,11]. By applying an A.C. voltage to an eDEP device, a large electric field gradient is generated around the fine electrodes, and this electric field gradient generates a DEP force that acts on cells. Because the electric field gradient determines the magnitude and direction of the DEP force acting on cells, it is necessary to generate within the device an electric field of adequate strength to manipulate target cells with high precision. However, cell separation devices based on dielectrophoresis have the drawback that the cells inside the device are exposed to a high-temperature environment due to Joule heating [12,13]. The degree of Joule heating mostly depends on the square of the electric field magnitude [14,15]. Thus, there is a risk that cells inside the device are exposed to a high-temperature environment due to the Joule heat generated by the application of high voltage [16,17].

It has been confirmed that high-temperature exposure may affect the physiological functionality of cells [18]. Moritz et al. conducted experiments using human and porcine epidermal cells and reported that irreversible cell damage occurs when the cells are exposed to a high-temperature environment above 44 °C for approximately 6 h [19]. Harmon et al. reported that apoptosis was significantly promoted by exposing murine mastocytoma cells to a high-temperature environment of around 44 °C [20]. Yamada et al. reported that cell division was halted when MKN-1 and SNG-M cells were exposed to a high-temperature environment of 42 °C [21]. Consequently, recent studies have focused on Joule heating and temperature fields within dielectrophoresis-based cell separation devices to evaluate their effects on cells [22,23,24]. In particular, the correlation between solution conductivity and the temperature increase has been extensively investigated. For example, Tay et al. measured the temperature field of planar electrodes using a NaCl solution with $\sigma_{f}=1$ S/m and reported a temperature rise of up to 100 °C [22]. Sridharan et al. conducted an observation experiment on fluid circulation within a device using a phosphate-buffered solution with $\sigma_{f}=4.7\times 10^{-2}$ S/m and predicted a temperature change of about 30 °C in their numerical simulations [23]. Nakano et al. measured the temperature field within an insulated DEP (iDEP) device using a phosphate-buffered solution with $\sigma_{f}=1.0\times 10^{-1}$ S/m and found that the temperature rose about 70 °C [24]. Among other relevant studies that measured the temperature rise due to Joule heating in microdevices, Williams et al. measured the temperature using the laser-induced fluorescence (LIF) method with varying solution conductivity and applied voltage [25]. Pramod et al. measured the nonuniform temperature profiles in a silicon microchannel heat sink due to Joule heating and flow maldistribution using the micro-LIF method [26,27].

Additionally, comparisons between experimentally observed and numerically simulated results of temperature field analysis in flow fields have been reported. For example, in one of the studies focused on experimental measurement, Erickson et al. measured the temperature rise due to Joule heating inside a three-dimensional microfluidic device made of poly (dimethylsiloxane) (PDMS) and glass and reported that the results matched the numerical simulations with an accuracy of ±3 °C [28]. Similarly, Williams et al. compared experimental results obtained using the LIF method with numerical simulation results and reported that the temperature rise was proportional to the square of the applied voltage [29].

With respect to the previous studies focused on numerical simulation, Tang et al. proposed a three-dimensional numerical simulation model of thermal fluid flow to investigate the correlation between Joule heating and temperature gradient focusing and reported that the numerical simulation results matched the experimental results well [30]. Rasmussen et al. conducted modeling to optimize flow sensors within microchannels and reported that the simulation results and the temperature field measurements using sensors based on the simulations matched with an error of less than 1% [31]. Many analyses of microflows have been performed using commercial software. Gallo et al. numerically predicted the temperature field due to Joule heating in an insulator-based DEP (iDEP) device [32]. Kwak et al. performed numerical simulations focusing on localized dielectric loss-induced heating in DEP devices and reported that the origin of the heating in DEP devices with electrically insulated electrodes was Debye relaxation [33]. Wang et al. numerically predicted the interactions between electrothermal flow and electroosmotic flow near the constriction region in the microchannel and clarified the temperature rise and flow field near the constriction [34]. Khoshmanesh et al. analyzed the temperature field in the unique DEP device they developed [35].

In this study, with the aim of studying the effects of high-temperature exposure on cells, the temperature rise in the microchannel of a previously developed eDEP cell separation device was evaluated experimentally and numerically [36]. For the experimental evaluation, the micro-LIF method was adopted to measure the temperature in the device. In the numerical approach, the thermal structure of the device was investigated by performing a numerical simulation in which the effect of heat generation due to a nonuniform electric field was incorporated in the thermofluidic model. Furthermore, parametric numerical simulations were performed with changes in the solution conductivity and sample solution flow rate, which affect cell separation performance. Finally, the correlation between the device operating conditions and temperature rise and their effects on cell viability were explored and discussed.

## 2. Thermofluidic Numerical Simulation

### 2.1. Thermofluidic Governing Equations

**Figure 1:**
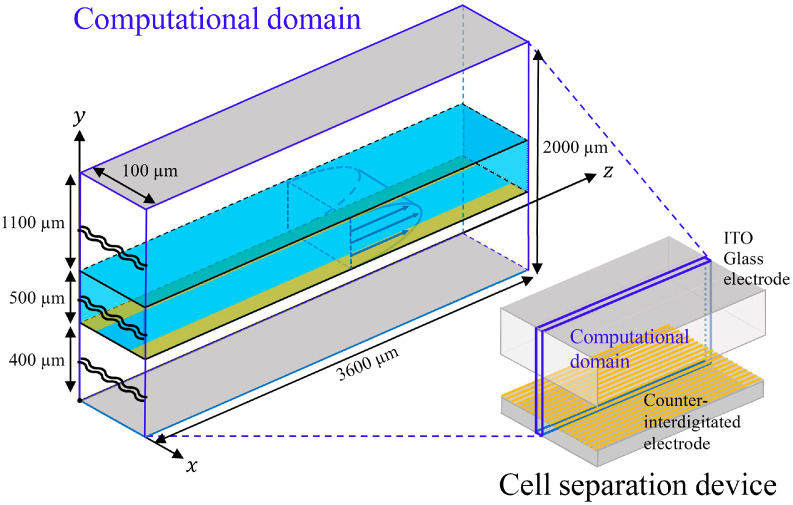
Conceptual diagram of the numerical simulation model.

Figure 1 shows a conceptual diagram of the numerical simulation model. The bottom surface of the microfluidic channel with height H=500 µm consisted of a 400 µm thick glass substrate with counter-interdigitated electrodes printed on it, and the top surface consisted of a 1100 µm thick indium tin oxide (ITO) planar electrode. The length of the microfluidic channel was $L_{z}=3600$ µm. Cell separation was performed by generating a nonuniform electric field while sample solution-suspended cells flowed through the microfluidic channel at a constant volumetric flow rate Q.

In the case of analyzing the flow of the electrolyte solution inside the microfluidic channel with the applied A.C. electric field, it was also necessary to evaluate the effects of electroosmotic flow and electrothermal flow near the electrode surfaces on the temperature field, in addition to Joule heating. It is known that the effect of the electroosmotic flow is likely to appear when the electric conductivity of the solution is $\sigma_{f}\leq 8.4\times 10^{-2}$ S/m [37]. Ren et al. reported that the order of magnitude of the electroosmotic flow was less than $1.0\times 10^{-6}$ m/s under the conditions of an applied voltage V=30 V_pp_ and conductivity $\sigma_{f}=1.0\times 10^{-3}$ S/m [38]. Electrothermal flows have been reported to be more pronounced when high-conductivity solutions ($\sigma_{f}\sim 0.2–1.0$ S/m) were used [37]. Akutsu et al. reported that the order of magnitude of electrothermal flow was about $7.5\times 10^{-5}$ m/s under conditions of an applied voltage V=20 V_pp_ and conductivity $\sigma_{f}=2.9\times 10^{-1}$ S/m [39]. In this study, an applied voltage of V=10 V_pp_ and conductivity of $\sigma_{f}=1.0\times 10^{-3}–1.0\times 10^{-1}$ S/m were adopted. Furthermore, taking into account that the average flow velocity of the sample solution was $U_{m}\sim \;0.5\times 10^{-3}$ m/s, the effects of electroosmotic and electrothermal flows on the thermofluidic field in the microchannel could be considered negligible. Regarding the buoyancy effect due to the temperature distribution in the microfluidic channel, the temperature difference in the microfluidic channel, ∆T, was only a few degrees Celsius, and the order of magnitude of the Rayleigh number, Ra, was Ra~100. Therefore, the buoyancy effect could also be considered negligible. The spanwise direction of the microchannel was defined as the x-axis, the height direction as the y-axis, and the flow direction as the z-axis. The sample solution was assumed to be an incompressible homogeneous viscous fluid. A fully developed Poiseuille flow was assumed for the velocity distribution. Thus, the velocity vector of the fluid was given as

(1) $$v=\left(0,\;0,\;\frac{\;6Q\;}{H^{3}}\left(H-y\right)y\right)$$

Here, H is the height of the microfluidic channel, and y is the height from the bottom surface (0≤y≤H). Q represents the volumetric flow rate of the sample solution, and it was given using the width of the microfluidic channel, B, as $Q=U_{m}BH$. The governing equations for the liquid and solid phases in the device are given as

(2) $$\;\frac{\;\partial T\;}{\partial t}+\left(v\cdot grad\right)\;T=\frac{\kappa_{f}}{{\;\rho }_{f}C_{pf}\;}\left(\frac{{\;\partial }^{2}T\;}{\partial x^{2}}+\frac{{\;\partial }^{2}T\;}{\partial y^{2}}+\frac{{\;\partial }^{2}T\;}{\partial z^{2}}\right)+\frac{q}{{\;\rho }_{f}C_{pf}\;}$$

(3) $$\;\frac{\;\partial T\;}{\partial t}=\frac{\kappa_{g}}{\;\rho_{g}C_{pg}\;}\left(\frac{{\;\partial }^{2}T\;}{\;\partial x^{2}}+\frac{{\;\partial }^{2}T\;}{\partial y^{2}}+\frac{\partial^{2}T\;}{\partial z^{2}}\right)$$

Here, the values t, κ, ρ, and $C_{p}$ are time, thermal conductivity, density, and isobaric specific heat, respectively. Subscripts f and g represent fluid and solid, respectively. The second term of the right-hand side of Equation (2) represents heat generation. The Joule heating $q=q\left(x,y\right)$ in the electrolyte solution can be given as

(4) $$q=\sigma_{f}E^{2}$$

where $\sigma_{f}$ is the electric conductivity of the electrolyte solution and E is the effective value of the electric field.

### 2.2. Thermofluidic Boundary Conditions

**Figure 2:**
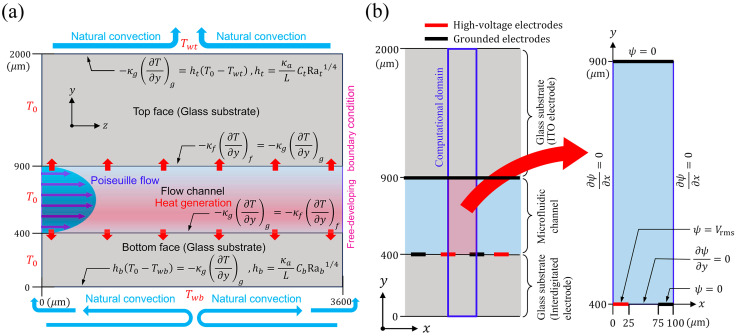
(**a**) Thermofluidic numerical simulation model. (**b**) Electric field analysis model.

The boundary conditions of the thermofluidic analysis are shown in Figure 2a. The constant temperature boundary condition $T=T_{0}$ was given for the upstream end of the device (z=0). The freely developed boundary condition of the temperature field was given at the downstream end of the device. The constant wall heat flux condition

(5) $$-\kappa_{f}{\left(\frac{\partial T}{\partial y}\right)}_{f}=-\kappa_{g}{\left(\frac{\partial T}{\partial y}\right)}_{g}$$

was applied to the interface between the liquid and the solid phases in the microfluidic channel. At the top surface of the device, the boundary condition

(6) $$-\kappa_{g}{\left(\frac{\partial T}{\partial y}\right)}_{g}=h_{t}\left(T_{0}-T_{wt}\right)$$

was applied. Here, $h_{t}$ is the convective heat transfer coefficient at the top surface, and $T_{wt}$ is the local temperature at the top surface. $h_{t}$ was evaluated using the following empirical formula:

(7) $$h_{t}=\frac{\kappa_{a}}{L}C_{t}{{Ra}_{t}}^{\zeta }$$

where L represents the characteristic length of the device and was defined as follows using the perimeter length, l, and area, A, of the electrode substrate:

(8) $$L=\frac{\;4A\;}{l}$$

The coefficients ζ and $C_{t}$ were determined empirically. ${Ra}_{t}$ is the Rayleigh number at the top surface, defined as

(9) $${Ra}_{t}=\frac{\;g\;\beta_{a}\;\left(T_{0}-T_{wt}\right)\;L^{3}\;}{{\nu_{a}}^{2}}{Pr}_{a}$$

where the values g, $\beta_{a}$, $\nu_{a}$, and ${Pr}_{a}$ are the gravitational acceleration, thermal expansion coefficient of the air, kinematic viscosity of the air, and Prandtl number of the air, respectively. At the bottom surface of the device, the boundary condition

(10) $$h_{b}\left(T_{0}-T_{wb}\right)=-\kappa_{g}{\left(\frac{\partial T}{\partial y\;}\right)}_{g}$$

was applied. Here, $h_{b}$ is the convective heat transfer coefficient at the bottom surface, and $T_{wb}$ is the local temperature at the bottom surface. In a similar manner as for $h_{t}$ in Equation (7), $h_{b}$ was evaluated using the following empirical formula:

(11) $$h_{b}=\frac{\kappa_{a}}{L}C_{b}{{Ra}_{b}}^{\zeta }$$

Here, ${Ra}_{b}$ is the Rayleigh number at the bottom surface, defined as

(12) $${Ra}_{b}=\frac{\;g\;\beta_{a}\;\left(T_{0}-T_{wb}\right)\;L^{3}\;}{{\nu_{a}}^{2}}{Pr}_{a}$$

The coefficients ζ and $C_{b}$ were determined empirically. For both lateral faces of the computational domain, the symmetry boundary condition

(13) $$\frac{\partial T}{\partial x}=0$$

was applied.

### 2.3. Governing Equation for Electric Field

The Joule heating per unit time, q, as expressed in Equation (4), is a function of the effective value of the A.C. electric field, E. Therefore, in this study, the electric field in the microchannel was obtained by electrostatic field analysis with E as the dependent variable. Initially, because fingers of the interdigitated electrodes were arranged along the flow direction (z-axis), the electric field in the microfluidic channel could be assumed to be uniform along the flow direction. In other words, the electric field only had a nonuniform distribution within the cross-section of the microfluidic channel. Therefore, the electric field in the microchannel was obtained by solving the Laplace equation for the electrostatic potential, ψ, in the xy-plane of the microchannel:

(14) $$\frac{\partial^{2}\psi }{\partial x^{2}}+\frac{\partial^{2}\psi }{\partial y^{2}}=0$$

The electrostatic field E (|E|=E) was obtained from the relation

(15) $$E=\left(E_{x}\;,\;E_{y}\right)=\left(-\frac{\;\partial \psi \;}{\partial x},-\frac{\;\partial \psi \;}{\partial y}\;\right)\;$$

using the ψ value obtained by numerically solving Equation (14).

### 2.4. Boundary Conditions for the Electric Field

Figure 2b shows the electric field analysis model. The computational domain was a part of the microfluidic channel cross-section, including half of a pair of interdigitated electrodes. The boundary conditions for ψ were given as

(16) $$\left\{\begin{array}{c} \;\;\psi =V_{rms}\;\left(High-voltage\;electrode\right)\;\;\\ \psi =0\;\;\;\;\;\;\;\left(Grounded\;electrodes\right)\;\;\;\;\;\;\; \end{array}\right.$$

The symmetry boundary condition

(17) $$\frac{\partial \psi }{\partial x}=0$$

was applied to both lateral faces of the computational domain (x=0, 100 µm). The boundary condition

(18) $$\frac{\partial \psi }{\partial y}=0$$

was applied to the bottom surface of the microfluidic channel (25 μm≤x≤75 μm, y=400 µm). The boundary condition

(19) $$E_{x}=0$$

was applied to the electrode surfaces and both lateral faces of the computational domain. The boundary condition

(20) $$E_{y}=0$$

was applied to the bottom surface of the microfluidic channel (25 μm≤x≤75 μm, y=400 µm).

### 2.5. Numerical Simulation Schemes

The governing equations and boundary conditions were discretized using the first-order accurate finite difference scheme in time and the second-order accurate scheme in space. For example, the discretized Equation (3) is denoted as

(21) $$T_{i,j,k}^{n+1}=\alpha_{0}\;T_{i,j,k}^{n}+∆t\left(\alpha_{1}T_{i+1,j,k}^{n+1}+\alpha_{2}T_{i-1,j,k}^{n+1}+\alpha_{3}T_{i,j-1,k}^{n+1}+\alpha_{4}T_{i,j+1,k}^{n+1}+\alpha_{5}T_{i,j,k-1}^{n+1}+{\alpha_{6}T}_{i,j,k+1}^{n+1}\right)$$

$$\left\{\begin{array}{c} \alpha_{0}=1+∆t\frac{2\kappa_{g}}{{\;\rho }_{g}{Cp}_{g}\;}\left(\frac{1}{\;∆x_{i}∆x_{i-1}\;}+\frac{1}{\;∆y_{j}∆y_{j-1}\;}+\frac{1}{\;∆z_{k}∆z_{k-1\;}}\right)\\ \alpha_{1}=\frac{2\kappa_{g}}{\;\rho_{g}{Cp}_{g}\;}\;\frac{1}{∆x_{i}\;\left(∆x_{i}+∆x_{i-1}\right)}\;\;\;\\ \alpha_{2}=\frac{2\kappa_{g}}{{\;\rho }_{g}{Cp}_{g}\;}\;\frac{1}{∆x_{i-1}\left(∆x_{i}+∆x_{i-1}\right)}\\ \alpha_{3}=\frac{2\kappa_{g}}{{\;\rho }_{g}{Cp}_{g\;}}\;\frac{1}{∆y_{j}\left(∆y_{j}+∆y_{j-1}\right)}\;\;\;\\ \alpha_{4}=\frac{2\kappa_{g}}{{\;\rho }_{g}{Cp}_{g}\;}\;\frac{1}{∆y_{j-1}\left(∆y_{j}+∆y_{j-1}\right)}\\ \alpha_{5}=\frac{2\kappa_{g}}{\;\rho_{g}{Cp}_{g\;}}\;\frac{1}{∆z_{k}\;\left(∆z_{k}+∆z_{k-1}\right)}\;\;\;\\ \alpha_{6}=\frac{2\kappa_{g}}{\;\rho_{g}{Cp}_{g\;}}\;\frac{1}{∆z_{k-1}\left(∆z_{k}+∆z_{k-1}\right)} \end{array}\right.$$

where $T_{i,j,k}^{n}$ is the temperature at grid point (i,j,k) in the computational domain at time t=n. Moreover, $∆x_{i}$, $∆y_{j}$, and $∆z_{k}$ are the distances to the adjacent grid points in the x, y, and z directions from the grid point (i,j,k), and ∆t is the time increment. The convection term in Equation (2) was discretized using the values from the previous time t=n.

**Figure 3:**
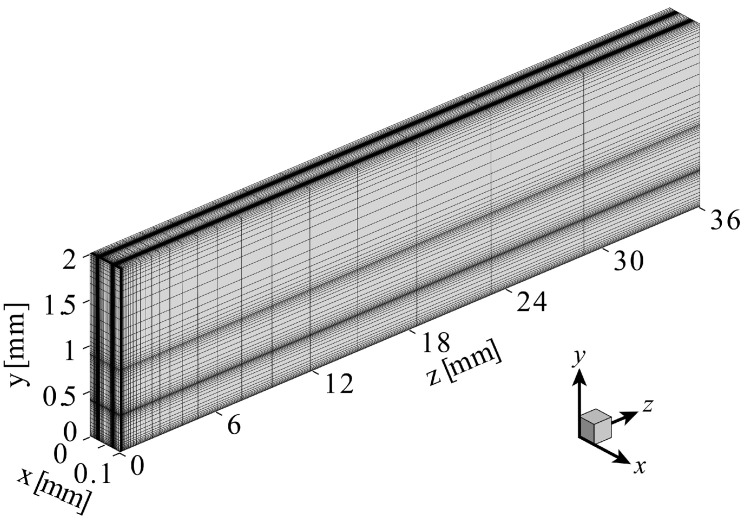
Example of the computational grid.

The computational grid is shown in Figure 3. Note that the number of grid points in the computational grid shown in the figure has been reduced for visibility. A finer grid system was adopted at the channel inlet, the interfaces between the fluid and the glass, and the interfaces between the glass and the air. The number of computational grid points used was ~1,300,000. This number of computational grids was the minimum number of grids that would not change the results of the simulation. For the heat transfer coefficient h of the horizontal plates corresponding to the upper and lower end surfaces of the device, many empirical formulas have been proposed in the past, and several of these are listed in Table 1. Regarding the exponent ζ of the Rayleigh number Ra in Equations (7) and (11), for the low Rayleigh numbers ($Ra\sim \leq 10^{5}$), which were relevant to the thermofluidic phenomena targeted in this analysis, a model with ζ~1/4 was proposed. Therefore, in this analysis, a model of $h\sim {Ra}^{1/4}$ was also adopted. Regarding the coefficients $C_{t}$ and $C_{b}$ appearing in Equations (7) and (11), their magnitudes were generally on the order of $10^{-1}$ [40,41,42,43]. Accordingly, the coefficients $C_{t}$ and $C_{b}$ for $h_{t}$ and $h_{b}$ were determined as

(22) $$\left\{\begin{array}{c} C_{t}=0.1\\ C_{b}=0.1 \end{array}\right.$$

To begin the numerical simulation for thermofluidic analysis, the electric field distribution was solved first using Equations (14)–(20). Then, using Equation (4), the distribution of q in the cross-section of the microfluidic channel was obtained. Next, using Equations (2)–(4), the temperature field in the device was obtained. Time integration was performed with a constant time step until the temperature at the bottom surface of the microfluidic channel became almost constant. The analysis conditions are listed in Table 2. The thermophysical properties used are listed in Table 3. In-house Fortran parallel-computing code managed using OpenMP library was used for the numerical simulations. Computations were performed on the multi-core parallel simulation system of the National Defense Academy.

## 3. Experimental

### 3.1. Device Fabrication

**Figure 4:**
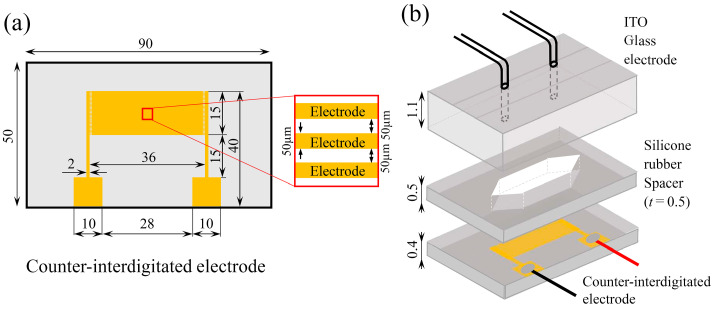
(**a**) Schematic of the electrode substrate. (**b**) Conceptual diagram of the components of the cell separation device.

Figure 4a shows the schematic of the electrode substrate. The counter-interdigitated electrode measured 36 mm long and 50 µm wide. The interdigitated electrode used in the experiment was an aluminum film with a thickness of 300 nm fabricated by standard photolithography. In brief, a glass plate measuring 50 mm×90 mm with a thickness of 0.4 mm was used as the electrode substrate. The plate surface was ultrasonically cleaned using acetone and isopropyl alcohol for 5 min each. A positive photoresist (S1805G; Rohm and Haas Electronic Materials) was spin-coated on the plate surface. The coated plate was baked at 90 °C for 3 min. The resist layer was exposed to UV light through a positive mask image. The exposed photoresist was developed and baked at 130 °C for 3 min. A 300 nm thick layer of the aluminum was vacuum-deposited over the plate. The uncovered aluminum area was etched with mixed acid at 40 °C for 90 s. Finally, the photoresist was removed using an AZ 100 remover (AZ Electronic Materials). Figure 4b shows the components of the cell separation device. The upper surface was a glass substrate with a thickness of 1.1 mm coated with ITO film. It had ϕ1.0 holes for introducing and draining the cell sample solution, which were made using a router (2307396; Sea Force). Polypropylene female Luer fittings were adhered to the perforated parts, and lead wires were attached to the electrode terminals using conductive epoxy adhesive. A microfluidic channel of dimensions 0.5 mm (H) × 36 mm ($L_{z}$) × 10 mm (B) was constructed with parallel top and bottom glass plates separated by 0.5 mm with a silicon rubber spacer.

### 3.2. Sample Solution Preparation

Rhodamine B (Sigma 83695-250MG; ex. 553 nm, em. 627 nm) was used as the fluorescent dye. A 300 mM mannitol solution was used as the solvent.

### 3.3. Experimental Setup

**Figure 5:**
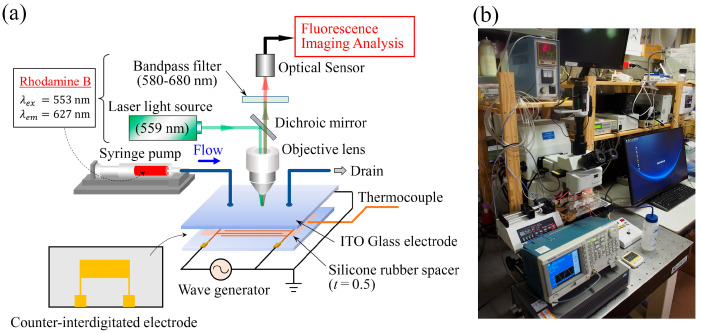
(**a**) Schematic diagram of the primary part of the experimental setup. (**b**) Snapshot of the experimental setup.

Figure 5 shows the schematic diagram of the main part of the experimental apparatus. The experimental apparatus is almost the same as the apparatus used in the cell separation experiment [36]. The width B and length X of the microfluidic channel were B=10 mm and X=36 mm, respectively. The temperature of the sample solution was measured with a T-type thermocouple. A 5 mL volume of sample solution was introduced into the microfluidic channel at a constant flow rate using a syringe pump, and an AC voltage of V=10 V_pp_ was applied to the device. A laser beam with an excitation wavelength of 559 nm and a bandpass filter with a wavelength range of 570–670 nm were used to acquire fluorescence images. Acquisition of a reference image for image division and measurement of the initial temperature were performed prior to applying the AC voltage to the device. Fluorescence image acquisition was performed every 30 s from the onset of the voltage application (t=0) until t=1500 s elapsed. The experimental conditions are listed in Table 4.

### 3.4. Image Processing

**Figure 6:**
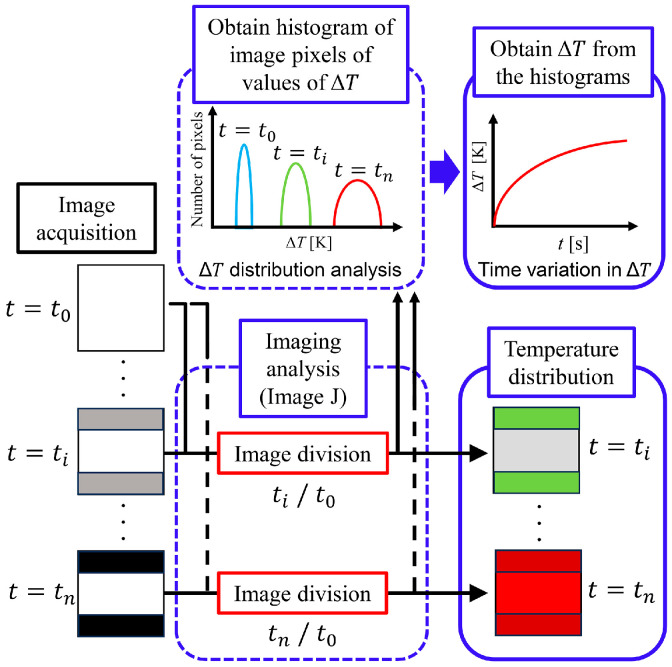
Procedure of the imaging analysis.

Figure 6 shows the procedure for converting the acquired fluorescence image into images of the distribution of temperature rise ∆T. ImageJ “https://imagej.nih.gov/ij/” (accessed on 1 November 2024) was used for image analysis. The time-lapse fluorescence images were sequentially read by ImageJ and divided by the reference image to produce images of the ratio of the fluorescence intensity. Values of the ratio of fluorescence intensity were converted into ∆T values using a calibration curve of temperature and fluorescence intensity ratio to obtain a pseudocolor temperature distribution. The mean value of ∆T at each elapsed time was obtained from the distribution of histograms of ∆T after temperature transformation, using Python3 (ver. 3.9.6) code.

### 3.5. Temperature Calibration

**Figure 7:**
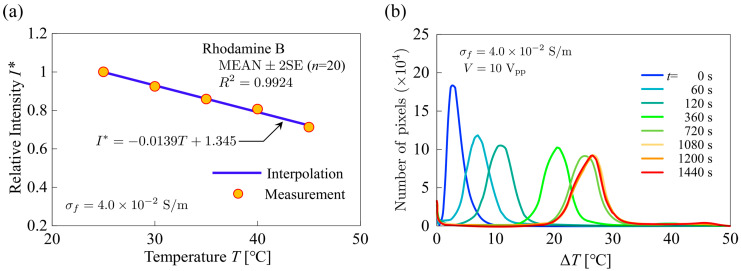
(**a**) Temperature calibration curve. (**b**) Histogram obtained by imaging analysis.

Calibration of the temperature against the fluorescence intensity ratio was performed using a plate reader (DTX-880; Beckman Coulter). The temperature in the chamber of the plate reader was changed every 5 °C from 25 °C to 45 °C to acquire the fluorescence intensity. The calibration curve obtained is shown in Figure 7a. The calibration curve was normalized to that at a temperature of 25 °C. The histogram obtained by imaging analysis is shown in Figure 7a. The average values of ∆*T* was defined as

(23) $$∆T=\frac{\sum_{p=1}^{M}{∆T}_{p}\cdot \;k\;\left({∆T}_{p}\right)}{\sum_{p=1}^{M}\;k\;\left({∆T}_{p}\right)}$$

where ${∆T}_{p}$ is the temperature rise of the p-th image pixel, k is the number of image pixels having the value of ${∆T}_{p}$, and M is the largest number of pixels. The resolution of the temperature rise was ${∆T}_{p}-{∆T}_{p-1}=0.26$ °C.

## 4. Results and Discussion

### 4.1. Developed Code Verification

#### 4.1.1. Developed Code Verification

**Figure 8:**
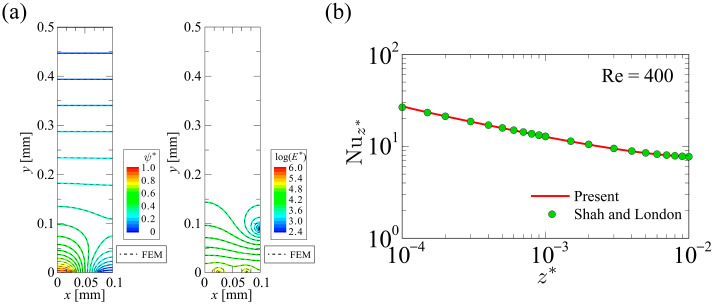
(**a**) Distributions of (left) electric potential and (right) electric field. (**b**) Local Nusselt number for isothermal wall.

Figure 8a shows the distribution of (left) the electrostatic potential, ψ*, and (right) the electric field, E*, in the cross section of the microfluidic channel. The potential and electric field are non-dimensionalized as $\psi *=\psi /{\;V}_{rms}$ and $E*=EH/{\;V}_{rms}$, respectively. The color contours represent the results using the developed simulation code, and dashed monochrome lines represent the results using the commercial software FEATool Multiphysics “https://www.featool.com/multiphysics/” (accessed on 1 November 2024). The number of grid points used in the commercial software was $2.6\times 10^{6}$. The convergence criterion of the relative error of the iterative calculation was defined as <${1.0\times 10}^{-8}$. As shown in Figure 8a, the electric field was extremely strong in the vicinity of the electrode edges. Thus, the strong electric field, which is highly localized in the microfluidic channel, acts as a strong local heat source. Therefore, it is essential to ensure sufficient spatial resolution in this region to accurately evaluate the temperature field. The electric field obtained by the developed simulation code was in good agreement with that obtained by the commercial software, confirming that the developed simulation code has sufficient accuracy. Figure 8b shows the profile of the local Nusselt number for parallel-plate laminar flow of isothermal walls obtained with the developed simulation code, compared with those reported by Shah and London [44]. The boundary conditions are constant wall temperature (T=23 °C) and constant inflow temperature ($T=T_{0}$). The dimensionless z-coordinate is defined as

(24) $$z^{*}=\frac{z}{2HRe\cdot Pr}$$

(25) $$Re=\frac{{\;2HU}_{m}}{\upsilon_{f}}$$

and calculations were performed under the condition of Re=400. The local Nusselt number is defined as

(26) $${Nu}_{z^{*}}=\frac{2H}{{\;T}_{w}\left(z^{*}\right)-T_{b}\left(z^{*}\right)\;}{\left(-\frac{\;\partial T\left(z^{*}\right)\;}{\partial y}\right)}_{y=0}$$

where $T_{w}$ is the temperature at the bottom surface of the microfluidic channel and $T_{b}$ is the bulk mean temperature of the fluid defined as

(27) $$T_{b}=\frac{\int_{S}{\;\rho }_{f}C_{pf}\;T\left(x,y,z^{*}\right)\;wdS}{\int_{S}{\;\rho }_{f}C_{pf}\;wdS\;}$$

where S represents the cross-sectional area of the microfluidic channel in the computational domain. The values of ${Nu}_{z^{*}}$ obtained by the developed simulation code were in good agreement with that obtained in the previous study, confirming that the developed thermofluidic simulation code has sufficient accuracy.

#### 4.1.2. Thermal Structure of the Device

**Figure 9:**
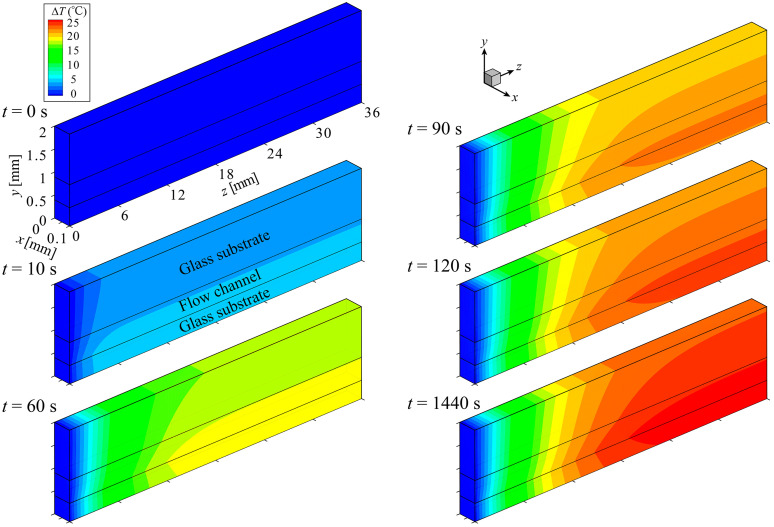
Transient of the temperature-rise distribution in the computational domain. ($\sigma =4\times 10^{-2}$ S/m, Q=5.0 mL/h).

Figure 9 shows the transient of the distribution of temperature rise, ΔT ($=T-T_{0}$), in the computational domain. The analysis conditions were set to $\sigma ={4.0\times 10}^{-2}$ S/m and Q=5.0 mL/h to match the experimental conditions of the developed cell separation device [36]. Numerical simulation was performed until t=1440 s when the cell separation of 2 mL of sample solution was completed in the experiment. ΔT reached its maximum value at the bottom of the microfluidic channel, where the generation of Joule heat was the greatest. The temperature distribution had a gradual increase along the flow direction (z) across the liquid phase (microfluidic channel) and solid phases (top and bottom glass substrates). The results imply that heat transport by diffusion is dominant over that by convection in the device. The maximum of ΔT appeared at the downstream end of the microfluidic channel, which was predicted to reach ~21 °C at time t=1440 s.

**Figure 10:**
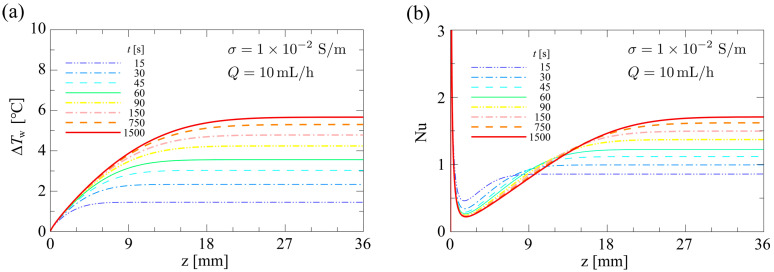
Time variations of the distributions of (**a**) the temperature rise at the bottom surface of the microfluidic channel and (**b**) the local Nusselt number.

Figure 10 shows the time variations of the distributions of (a) the temperature rise at the bottom surface of the microfluidic channel, $∆T_{w}$ ($=T_{w}-T_{0}$), along the flow direction, and (b) the local Nusselt number, Nu, along the flow direction, up to t=1500 s, for $\sigma =1.0\times 10^{-2}$ S/m and Q=10.0 mL/h. Nu is defined as

(28) $$Nu=-\frac{2H}{{\;T}_{w}\left(z\right)-T_{b}\left(z\right)\;}{\left(-\frac{\;\partial T\left(z\right)\;}{\partial y}\right)}_{y=0}$$

Here, ${\;T}_{w}$ and $\partial T\left(z\right)/\partial y$ are the values of the local temperature and temperature gradient at the bottom of the microfluidic channel, averaged with respect to the span direction.

(29) $$\;T_{w}\left(z\right)=\frac{\int T_{w}\left(x,0,z\right)dx}{\int dx\;}\;,\;\;{\left(\frac{\;\partial T\left(z\right)\;}{\partial y}\right)}_{y=0}=\frac{\int {\left(\partial T\left(x,y,z\right)/\partial y\right)}_{y=0}dx}{\int dx\;}$$

Unless otherwise specified, all distributions of thermophysical quantities in the flow direction represent the spanwise averaged distribution. As shown in Figure 10a, because the Joule heat was generated almost uniformly along the flow direction over the bottom surface of the microfluidic channel, $\Delta T_{w}$ began to increase uniformly in the flow direction as soon as the voltage was applied. On the other hand, the upstream end of the microfluidic channel was cooled by the inflow fluid. Therefore, while $\Delta T_{w}$ increased uniformly with time, a distribution was presented in which the high-temperature plateau gradually narrowed from the upstream side due to the cooling effect of the convection heat transport of the inflow fluid. As shown in Figure 10b, the area near the inlet of the microfluidic channel, ${\;T}_{w}-T_{b}$, presented its maximum value due to Joule heating, and the Nusselt number, Nu, presented its minimum value. On the other hand, $T_{b}$ did not increase because the area near the inlet continued to be cooled. Therefore, the minima of Nu became even smaller as time passed. On the downstream side of the microfluidic channel, Nu gradually increased uniformly due to Joule heating. Compared to the distribution of Nu shown in Figure 8b, which was obtained under the condition of constant wall temperature, the distribution obtained in this study was different. In the thermofluidic field under study, the fluid in the microfluidic channel was found to be heated spatially nonuniformly by Joule heating due to the presence of the nonuniform electric field. These are the characteristics of the temperature field within the cell separation device.

This analysis did not take into account the presence of cells in the microfluidic channel. No quantitative assessment has been reported regarding the potential impact of cells present in the solution on the temperature generated by DEP. Given that 60–70% of a cell’s composition is water, it is expected that the thermal properties of cells will not significantly deviate from those of the solution. Therefore, it is considered unlikely that the presence of cells in the solution will affect the surrounding thermal environment. On the other hand, when selecting a solvent, the solvent should have the property of mitigating the temperature rise in the microchannel. For example, certain organic solvents or oils have been reported to have a solution conductivity $\sigma_{f}=10^{-6}–10^{-10}\;S/m$ at 20 °C, which is expected to help mitigate temperature rise due to Joule heating. However, cautious discussion is necessary when considering the use of these types of solutions, as they may pose various concerns regarding potential impacts on the physiological functions of cells.

#### 4.1.3. Influence of Flow Rate and Conductivity on Temperature

**Figure 11:**
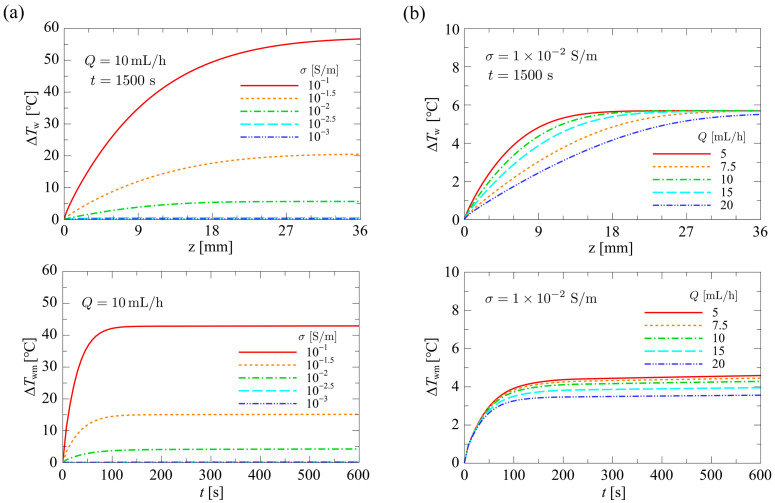
(**a**) σ dependence of (top) the distribution of the temperature rise and (**bottom**) the time variation of the mean temperature rise. (**b**) Q dependence of (**top**) the distribution of the temperature rise and (bottom) the time variation of the mean temperature rise.

Figure 11 shows (top) the distribution of the local temperature rise at the bottom surface of the microfluidic channel, $\Delta T_{w}$, along the flow direction and (bottom) the time variation of the mean temperature rise, $\Delta T_{wm}$, for varying σ and Q. $\Delta T_{wm}$ is defined as

(30) $$\Delta T_{wm}\left(t\right)=\frac{\int \Delta T_{w}\left(t\right)dz}{\int dz\;}\;$$

For $\Delta T_{w}$, the distribution for the thermal steady state (t=1500 s) is shown, and for $\Delta T_{wm}$, the transient variation is shown up to t=600 s, when the value changed significantly. In Figure 11a, Q was fixed at Q=10.0 mL/h, and σ was varied in the range $\sigma =1.0\times 10^{-3}–1.0\times 10^{-1}$ S/m. In Figure 11b, σ was fixed at $\sigma =1.0\times 10^{-2}$ S/m, and Q was varied in the range Q=5.0–20.0 mL/h. As shown in the top panel of Figure 11a, the distribution of $\Delta T_{w}$ increased proportionally with σ. This is because the heat generation term q on the right-hand side of Equation (4) is proportional to σ. As shown in the top panel of Figure 11b, the effect of convective heat transfer became significant with increasing Q, and the shape of the $\Delta T_{w}$ profile transitioned from a parabolic form to a linear form. From the above, it was found that Q is the dominant parameter for the shape of the distribution of $\Delta T_{w}$ in the microfluidic channel. Therefore, in order to maintain a uniform temperature distribution in the microfluidic channel, which is desirable for cell separation devices, it is essential to minimize the value of Q as much as possible. In this study, the heat exchange by conduction was not considered as a thermal boundary condition at the downstream end of the device. Therefore, under actual cell separation operations, the temperature at the downstream end of the device is expected to be affected by the external thermal environment at the downstream end of the device.

**Figure 12:**
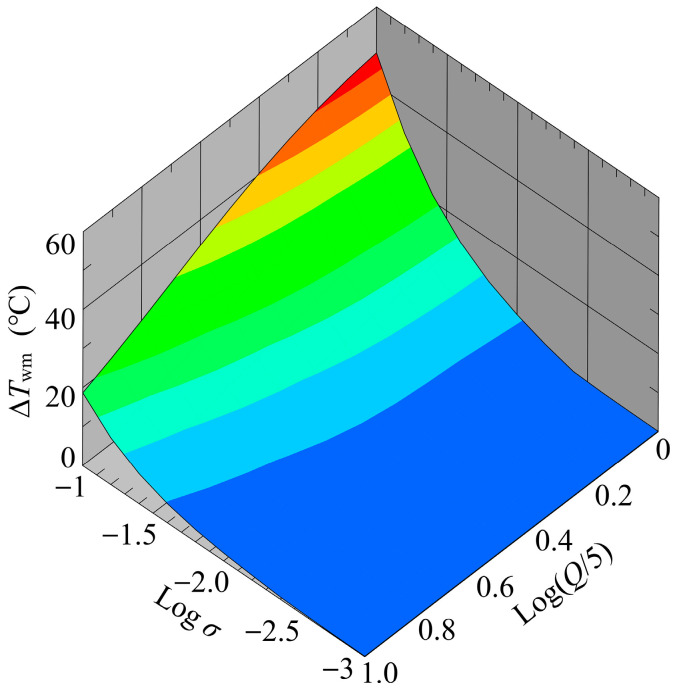
Variation of the mean temperature rise with respect to variations of flow rate and conductivity.

Figure 12 shows a bird’s-eye view of $\Delta T_{wm}$ as a function of both σ and Q after the temperature field reached a steady state. Parametric calculations (~200 simulations) were performed with σ varying in the range of $\sigma =1.0\times 10^{-3}-1.0\times 10^{-1}$ S/m, and Q in the range of Q=5.0–50.0 mL/h. As shown in Figure 12, the value of $\Delta T_{wm}$ changed rapidly in the range of $\sigma =1.0\times 10^{-2}–1.0\times 10^{-1}$ S/m regardless of the value of Q. Moreover, at higher values of σ, it was found that the value of $\Delta T_{wm}$ varied significantly with variations in Q. Under these conditions, the effect of convective heat transport on the temperature rise was pronounced due to the high heat generation, resulting in a distinct Q-dependence of $\Delta T_{wm}$. The maximum value of $\Delta T_{wm}$ reached approximately 48 °C. Elengoe et al. reported that the viability of MCF10A and MDA-MB-231 cells was decreased by exposure to a 41 °C environment for 30 min [45]. Furthermore, it has been reported that exposure to high-temperature environments induces adverse effects on cells, including cell death and reduced viability [19,20,21]. Therefore, the highest value of the predicted $\Delta T_{wm}$ is considered to have a non-negligible influence on cells during the cell separation process.

#### 4.1.4. Influence of Flow Rate and Conductivity on the Nusselt Number

**Figure 13:**
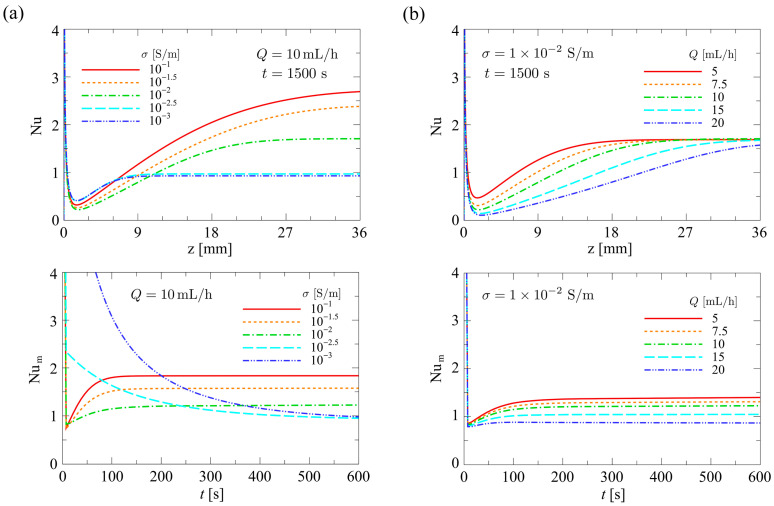
(**a**) σ dependence of (top) the distribution of the Nusselt number and (**bottom**) the time variation of the mean Nusselt number. (**b**) Q dependence of (**top**) the distribution of the Nusselt number and (bottom) the time variation of the mean Nusselt number.

Figure 13 shows (top) the distribution of the local Nusselt number at the bottom surface of the microfluidic channel, Nu, along the flow direction and (bottom) the time variation of the mean Nusselt number, ${Nu}_{m}$, for varying σ and Q. ${Nu}_{m}$ is defined as

(31) $${Nu}_{m}\left(t\right)=\frac{\int Nu\left(t\right)dz}{\int dz\;}\;$$

For Nu, the distribution for the thermal steady state (t=1500 s) is shown, and for ${Nu}_{m}$, the transient variation is shown up to t=600 s, when the value changed significantly. In Figure 13a, Q was fixed at Q=10.0 mL/h, and σ was varied in the range of $\sigma =1.0\times 10^{-3}–1.0\times 10^{-1}$ S/m. In Figure 13b, σ was fixed at $\sigma =1.0\times 10^{-2}$ S/m, and Q was varied in the range of Q=5.0 ~ 20.0 mL/h. As shown in the top panel of Figure 13a, the distribution of Nu exhibited its minimum value near the inlet of the microfluidic channel regardless of the value of σ. Then, due to the heat balance between Joule heating and heat diffusion from the bottom surface of the microfluidic channel, Nu gradually increased along the flow and eventually asymptotically approached a constant value. The position from the inlet of the microfluidic channel where Nu asymptotically approached a constant value moved downstream in proportion to the value of σ, i.e., the amount of heat generated. In other words, the value of z at which the temperature field reaches thermal equilibrium shifted downstream as σ increased. In the bottom panel of Figure 13a, for a large value of σ, ${Nu}_{m}$ exhibited a minimum value for a short while (<~20 s) after the onset of the voltage application. This is because, in cases of large heat generation, the $T_{b}$ was initially low, resulting in a large $T_{w}-T_{b}$ near the inlet. As the temperature rose, heat dissipation from the bottom surface of the microfluidic channel increased, causing ${Nu}_{m}$ to gradually increase and eventually reach a constant value when the device reached thermal equilibrium. The top panel of Figure 13b shows the dependence of the Nu distribution on Q. As the value of Q increased, the value of Nu decreased. This is because the temperature rise in the microfluidic channel was suppressed due to the inflow of fluid at room temperature from the inlet. In this study, heat conduction at the downstream end of the microfluidic channel is not considered in the thermal boundary condition. Therefore, ${Nu}_{m}$ decreased simply in accordance with the increase in Q as shown in the bottom panel of Figure 13b. The convective heat transport due to the increase in Q would suppress the temperature rise in the microfluidic channel.

**Figure 14:**
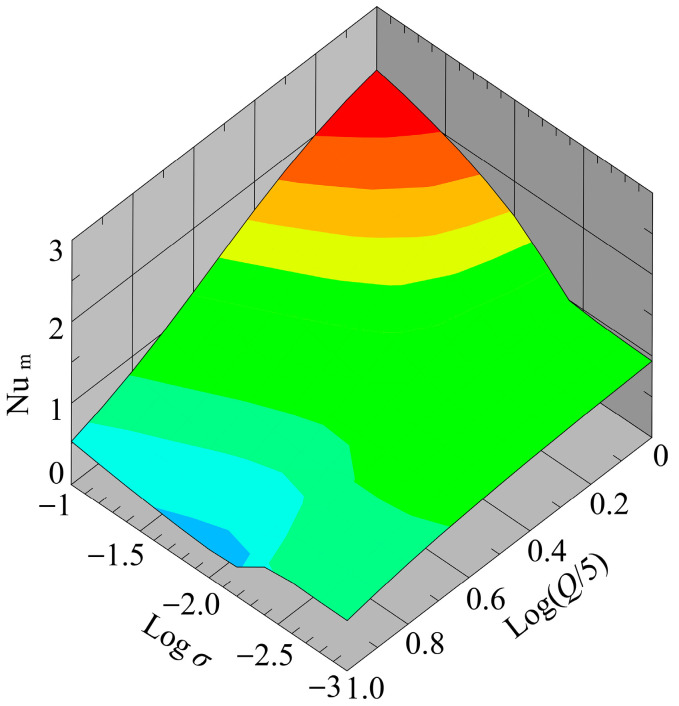
Variation of the mean Nusselt number with respect to variations in flow rate and electric conductivity.

Figure 14 shows a bird’s-eye view of ${Nu}_{m}$ as a function of both σ and Q after the temperature field reached a thermal steady state. In the range of $\sigma =1.0\times 10^{-2.2}–1.0\times 10^{-1}$ S/m, ${Nu}_{m}$ transitioned smoothly with changes in Q, while in the range of $\sigma =1.0\times 10^{-3}–1.0\times 10^{-2.2}$ S/m, ${Nu}_{m}$ remained nearly flat regardless of the value of Q. The maximum value of ${Nu}_{m}$ appeared when σ was at its maximum and Q was at its minimum, while the minimum value of ${Nu}_{m}$ appeared when $\sigma =1.0\times 10^{-2.2}$ and Q=50.0 mL/h. The results suggest that $\sigma \sim 1.0\times 10^{-2.2}$ S/m may be the threshold for a significant change in the thermal structure in the microfluidic channel of the present cell separation device.

### 4.2. Experimental Results and Comparison with Numerical Simulation

**Figure 15:**
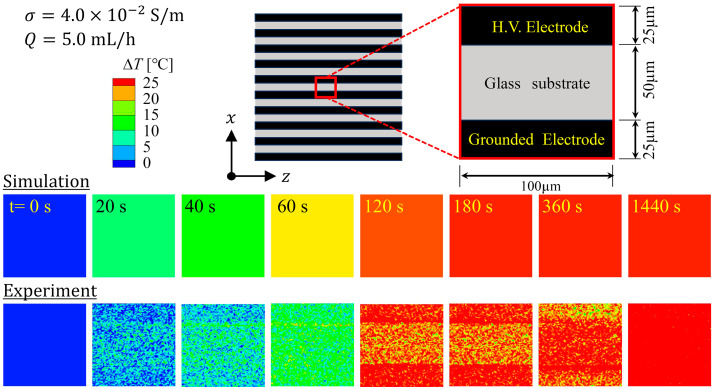
Transient of the temperature-rise distribution at the bottom surface of the microfluidic channel. Numerical simulation results (**top**) and experimental results (**bottom**).

**Figure 16:**
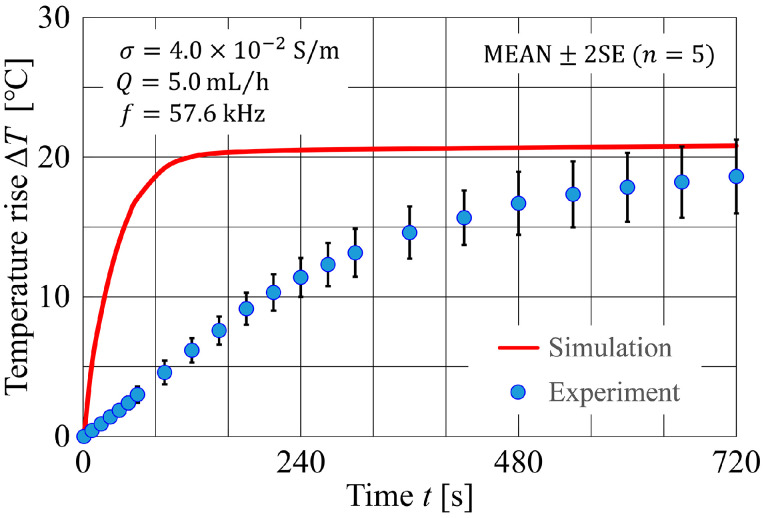
Time variation of the average temperature rise at the bottom of the microfluidic channel.

Figure 15 shows the transient of the distribution of the temperature rise, ∆T, at the bottom surface of the microfluidic channel within a 100 μm×100 μm area in the center of the device, displayed in pseudocolor. The top panel represents the numerical simulation results, and the bottom panel represents the experimental results. In the numerical simulation, ∆T increased significantly until t≅120 s, after which it hardly increased at all. In contrast, the experimental results showed a gradual increase in ∆T up to t≅1440 s. Moreover, the experimental results showed some temperature difference between the electrode and glass substrate, whereas the simulation results showed uniform ∆T distributions. This difference may be attributable to the fact that the numerical simulation model ignored the thickness of the metal electrode (300 nm). The difference in heat capacity between the metal and glass may be responsible for the difference in the rate of temperature rise. However, since heat transport by conduction was dominant in the microfluidic channels, there was actually little temperature difference between the electrode surface and the glass surface, as shown in the experimental results. Figure 16 shows the transient of the averaged ΔT with respect to the 100 μm×100 μm area in the center of the bottom surface of the microfluidic channel, comparing the experimental and numerical simulation results. In the experiment, as can be seen from the transient behavior of ΔT in Figure 16, the temperature field in the device nearly reached a steady state at t≅720 s, whereas in the numerical simulation, it reached a steady state at t≅240 s. The difference in the time history of ΔT between the two may be due to the fact that the numerical simulation did not take into account the effect of heat dissipation from both lateral sides of the device (relative to the flow direction). To evaluate the impact of heat loss from both sides of the device, numerical simulations using a model that isolates the side portions of the device may be considered. Additionally, adopting a model that accounts for heat conduction at the downstream end of the device is expected to allow a more accurate assessment of the temperature profile. Alternatively, simulations using a bulk model that ignores the interdigitated electrode structure may be considered as a more practical option. Another factor to consider is the validity of the values of $h_{b}$ and $h_{t}$. In this study, the values of $h_{b}$ and $h_{t}$ were estimated using reasonable empirical formulas that took into account the length scales under analysis and were roughly of the order of magnitude of 2–3 digits [46]. On the other hand, in previous reports on numerical simulation of Joule heating within microfluidic channels [23,24], the value of the heat transfer coefficient h was significantly higher than what is typically expected from natural convection of air, covering a range of 20–20,000 W/(m^2^⋅K). A value of h~20,000 W/(m^2^⋅K) is on the same order of magnitude as that observed in boiling heat transport phenomena. The setting of various parameter values used for boundary conditions could potentially have a non-negligible effect on the evaluation of the temperature field. Nakano et al. [24] have pointed out that the results of temperature field analysis through numerical simulation largely depend on the determination of h values. In modeling for thermofluidic analysis, it is believed that physically reasonable assumptions are crucial for constructing models that contribute to the accurate understanding of phenomena. Regarding the accuracy and error assessment of the micro-LIF method, a previous study has shown that an error of approximately ± 2 °C can occur at a 95% confidence level [47]. In the present study, the measurement error was approximately ± 2.5 °C up to t=720 s as shown in Figure 16. It was also confirmed that measurement error increased over time, which is thought to be attributable to electrode degradation caused by prolonged high electric field application. This effect would be mitigated by reducing the applied field intensity and the duration of application. Therefore, the measurement error is expected to decrease even when the temperature differentials are small. The other factors contributing to error include the fluctuations in the luminance of the laser sheet due to the scanning of the laser beam and intensity noise, which are expected to result in greater measurement errors compared to conventional LIF methods. Systematic error evaluation of the micro-LIF method in this study is challenging. However, with the introduction of methods such as the two-color LIF technique, more accurate measurements may be achievable in the future. The impact of measurement temperature error could have non-negligible adverse effects on the physiological functions of cells if, for instance, there is a 2 °C increase from the average experimental result. This is because even a few degrees of variation in a high-temperature environment above 40 °C can induce significant adverse effects on cellular physiological functions.

## 5. Conclusions

In recent years, dielectrophoresis-based cell separation technologies have attracted attention as a method that is noninvasive to cells. On the other hand, there are concerns about the effects of the high-temperature environment inside the device caused by Joule heating due to the strong electric field on the physiological functions of cells. In this study, we investigated the temperature field inside the microfluidic channels of a previously developed cell separation device through both experimental and numerical approaches. In the experiment, the micro-LIF method was adopted to measure the temperature in the device. In the numerical approach, the thermal structure of the device was investigated by performing a numerical simulation in which the effect of heat generation due to a nonuniform electric field was considered in the model. The experimental results revealed that the temperature rise at the bottom surface of the microfluidic channel was approximately 20 °C after 1440 s of voltage application. The transient of the thermofluidic phenomena in the device was investigated by numerical simulation, and an attempt was made to elucidate the thermal structure of the device. Additionally, a parametric analysis was conducted to investigate the correlation between the flow rate of the cell sample solution or the solution conductivity and the temperature field. The results suggested that, under certain operational conditions, the temperature rise from room temperature could exceed 40 °C. Our findings suggested that under certain operating conditions, the device could significantly affect the physiological functions of cells. Due to the structure of the proposed device, electric field lines concentrate at the edges of the electrodes. Since the solution near this region acts as a heat source, it may be challenging to reduce the heat generation itself. Moreover, considering the thermal transport characteristics at the microscale, attempts to mitigate the overall temperature increase of the device may be effective, as heat generated within the microchannel diffuses efficiently throughout the device. Methods to suppress the temperature rise of devices include using metal, which has higher thermal conductivity than glass, as the material for the device’s upper electrode plate and inducing forced convection of air on the substrate surface. It is believed that these approaches enhance the heat dissipation of the device. Additionally, leveraging the microscale advantage to further miniaturize the device may enhance heat transfer. However, verifying these effects is challenging, as measuring temperature within microchannels by methods other than optical techniques, such as LIF methods, is extremely difficult. Thus, at present, accurately predicting the temperature rise may rely solely on high-precision numerical simulations. To achieve both an improvement in device performance and a reduction in stress for the cells, detailed exploration of the optimal operating conditions is indispensable.

## Figures and Tables

**Table 1:** Empirical formulas proposed for the convective heat transfer coefficient h.

Range of Ra	Empirical Formula	Refs.
$10^{4}\leq Ra\leq 10^{7}$ (Top)	$h=0.54{Ra}^{1/4}$	McAdams et al. [40]
$10^{5}\leq Ra\leq 2\times 10^{7}$ (Top) ${3\times 10}^{5}\leq Ra\leq 3\times 10^{10}$ (Bottom)	$h=0.54{Ra}^{1/4}$ $h=0.27{Ra}^{1/4}$	Fishenden et al. [41]
$2\times 10^{5}\leq Ra\leq 4\times 10^{7}$ (Top)	$h=0.70{Ra}^{1/4}$	Al-Arabi et al. [42]
$1\times 10^{2}\leq Ra\leq 6\times 10^{3}$ (Top)	$h=0.297{Ra}^{1/4}$	Yousef et al. [43]

**Table 2:** Values of parameters used in the numerical simulation.

Parameters	Values
Number of grid points	1,300,000
Time increment∆t [s]	$1.0\times 10^{-2}$
Characteristic length L [m]	${6.0\times 10}^{-2}$
$\mathrm{Characteristic}\;\mathrm{temperature}\;T_{0}$ [K]	293
$\mathrm{Effective}\;\mathrm{potential}\;V_{rms}$ [V]	$5/\sqrt{2}$
$\mathrm{Solution}\;\mathrm{conductivity}\;\sigma_{f}$ [S/m]	$1.0\times 10^{-3}-1.0\times 10^{-1}$
Flow rate Q [mL/h]	5.0–50.0

**Table 3:** Thermophysical properties used in the numerical simulation (20 °C, 1 atm).

	κ [W/(m⋅K)]	ρ [kg/m^3^]	$C_{p}$ [J/(kg⋅K)]	ν [m^2^/s]	β [1/K]	Pr
Water	${6.03\;\times \;10}^{-1}$	$9.97{\;\times \;10}^{2}$	$4.19{\;\times \;10}^{3}$	$1.00\;\times \;10^{-6}$	$0.20\;\times \;10^{-3}$	7.01
Air	$2.57{\;\times \;10}^{-2}$	1.17	${1.01\;\times \;10}^{3}$	$1.50\;\times \;10^{-6}$	$3.41\;\times \;10^{-3}$	0.71
Glass	1.05	$2.20{\;\times \;10}^{3}$	${8.40\;\times \;10}^{2}$	―	―	―

**Table 4:** Experimental conditions.

Parameters	Values
Applied voltage V$\;[V_{pp}$]	10
Rhodamine B solution [μM]	20
$\mathrm{Solution}\;\mathrm{conductivity}\;\sigma_{f}$ [S/m]	$4.0\times 10^{-2}$
Frequency f [kHz]	57.6
Flow rate Q [mL/h]	5

## Data Availability

Data will be provided on reasonable request.
